# A Case of Eclampsia

**Published:** 1854-08

**Authors:** J. V. Withers

**Affiliations:** Brandenburg, Ky.


### ART. III.—A CASE OF ECLAMPSIA.

BY J. V. WITHERS, M. D., OF BRANDENBURG, KY.

As the condition mentioned above is very rare, this being the first case I have met with in a practice of more than eight years, I thought I would report the same, not with the view of benefiting any one with what I did on the occasion, but hoping I may make some reflections that will not be entirely devoid of interest, and perhaps profit, if such a case should fall into the hands of some of the younger members of the profession.

The best observed statistics on the subject confirms the fact, that more than one half the mothers and nearly all the children, die of eclampsia, when attacked during labor.

The patient was a negro girl, aged twenty-two years, primipara, stout, athletic, had been subject to convulsions when a child; I saw her at about 1½ o'clock, P. M., July 16th, where I found in attendance an old lady, from whom I learned the following facts. That she was taken with premonitory labor pains about 2 o'clock, A. M., which progressed regularly and naturally, with the exception of at times, an expression by the patient, of impatience, restlessness, and foreboding of evil to happen to her during her confinement. But by diverting her attention she would become lively and talkative. By 10 o'clock, the child had made considerable progress in descent, ready to engage in the inferior strait. Uterine orifice pretty well dilated, she anticipated a speedy termination of the labor. The pains had not been, and were not now, unusually severe, although she had been for the last hour or two more unmanageable. About this time she was attacked with a convulsion, lasting from one to two minutes, followed by a complete aberration of the mental faculties, not being able to recognise her parents; they returned every thirty or forty minutes with increased force, when I arrived she had had some three or four. The labor pains had nearly or quite ceased, at least they were feeble and quite inefficient, had been so from the first convulsion; when I arrived it was at about the termination of an interval. The expression of the countenance and eyes, was wild and delirious, incoherent talking, pulse full and about ninety beats per minute, skin hot. She seemed acutely sensitive to the touch about the genital organs, so much so, that it was with difficulty I could effect an examination, per vaginam. I found the breech of a child engaging in the inferior strait, with the orifice pretty well dilated. She immediately went off into a convulsion, eyes partly opened, rolled upward and to the right side; upper and lower extremities rigidly extended, frothing at the mouth, uterine walls flaccid with their contents falling to either side the most prone; this lasted about a minute, went off with an irregular twitching of the muscles of the face and extremities, succeeded by a loud mucous *râle*, no disposition to change her position during the convulsion, somnolency greater than when I first arrived, with great difficulty that she can be aroused, pays no attention to surrounding spectators. I immediately opened a vein in the arm and permitted it to run as long as I thought it safe, had cloths wrung out of cold water applied to the scalp, sinapism down the spine, and permitted the fresh air to play on her freely. It was with great difficulty that anything could be administered by the mouth, she did not seem to comprehend the art of swallowing. An hour intervened before another convulsion; I gave her chloroform by inhalation, at its outset, which rendered it light compared with the one before. Before she became so she would to some extent heed what was spoken to her, and expressed a willingness to drink some cold water, which she did. There being an entire absence of uterine contractions, and the uterine orifice being dilatable and not sufficiently dilated to take the child away with the hand, I gave from ten to twenty drops oil of ergot every fifteen minutes, which, in the course of an hour, produced two pains—which fully engaged the child in the inferior strait and entirely dilated the orifice, the pains then subsided, and never returned again. The convulsions recurring, I subjected her to a second venesection, which resulted in no material benefit, the convulsions still occuring at intervals of an hour. The chloroform would mitigate and cut them short, but not arrest them; my efforts had been to arrest the convulsions. Believing this to be impossible, my forceps having come at 6½ o'clock, I determined on delivering her. I knew this was the last resort, though a hopeless one to me, so far as it concerned saving her life, for as soon as I arrived and saw the character of her convulsions, and the extent to which they had gone, I felt convinced they would prove fatal, and so expressed myself to the family, but a physician should do his whole duty. So I proceeded to apply the forceps—I am aware that the majority of writers on obstetrics, oppose their use in pelvic presentations, yet, believing the child to be dead, I felt that one objection was removed, and the admission of M. CAZEAUX, that M. STOLTZ recommended them, and that M. P. DUBOIS would not hesitate to use them under such circumstances, I felt fully justified, and accordingly succeeded in delivering the child in a few minutes, without the forceps slipping or giving way in the least. On passing my hand into the uterine cavity, I found a second child, above the superior strait, vertex presenting. I introduced the forceps and by 7½ o'clock, had succeeded in delivering the second and the placenta, and put the woman to bed.

Both the children were dead, and no doubt had been for some time; I ordered frequent doses of calomel, turpentine and castor oil. She could not swallow; the convulsions continued, she became entirely comatose, with stertorous breathing, and at 8½ o'clock died.

*Reflections*. 1*st*. No doubt but by 8 o'clock, A. M., the premonitory eclamptic symptoms were on her, when she ought to have been subjected to proper treatment, whereas six hours intervened before any was had, and four hours from actual convulsions, which had made such serious inroads on the system, that any treatment would have been unavailing.

*2nd.* The convulsions were of the gravest character, having proved fatal in ten and a half hours from their invasion, whereas their usual termination is in from twelve to forty-eight hours.

*3rd.* The entire suspension of uterine contractions in such cases is rarely met with, they continuing throughout.

*4th.* From the partial good effects manifested from the use of the chloroform, if it could have been used in conjunction with blood-letting at 8 o'clock, or 10 o'clock, A. M., the results might have been different. In this way combining, the sedative, anti-spasmodic depletory treatment of M. CHAILLY and others, early in the attack, with better prospects of success, and it seems from M. CHAILLY'S showing that this form of treatment has been more successful than the purely depletory, only one-fourth proving fatal under the former, and one-half under the latter.

